# Imidazoquinoxaline anticancer derivatives and imiquimod interact with tubulin: Characterization of molecular microtubule inhibiting mechanisms in correlation with cytotoxicity

**DOI:** 10.1371/journal.pone.0182022

**Published:** 2017-08-10

**Authors:** Alexis Courbet, Nicole Bec, Caroline Constant, Christian Larroque, Martine Pugniere, Safia El Messaoudi, Zahraa Zghaib, Sonia Khier, Carine Deleuze-Masquefa, Florence Gattacceca

**Affiliations:** 1 University of Montpellier, Montpellier, France; 2 Department of Biochemistry & Institute for Protein Design, University of Washington, Seattle, WA, United States of America; 3 IRCM, Institut de Recherche en Cancérologie de Montpellier, Montpellier, France; 4 IRCM, Institut de Recherche en Cancérologie de Montpellier INSERM U1194, Montpellier, France; 5 Institut Régional du Cancer de Montpellier, Montpellier, France; 6 IBMM, Institut des Biomolécules Max Mousseron, UMR 5247, Université de Montpellier, Montpellier, France; Duke University School of Medicine, UNITED STATES

## Abstract

Displaying a strong antiproliferative activity on a wide variety of cancer cells, EAPB0203 and EAPB0503 belong to the imidazo[1,2-*a*]quinoxalines family of imiquimod structural analogues. EAPB0503 has been shown to inhibit tubulin polymerization. The aim of the present study is to characterize the interaction of EAPB0203 and EAPB0503 with tubulin. We combine experimental approaches at the cellular and the molecular level both *in vitro* and *in silico* in order to evaluate the interaction of EAPB0203 and EAPB0503 with tubulin. We examine the influence of EAPB0203 and EAPB0503 on the cell cycle and fate, explore the binding interaction with purified tubulin, and use a computational molecular docking model to determine the binding modes to the microtubule. We then use a drug combination study with other anti-microtubule agents to compare the binding site of EAPB0203 and EAPB0503 to known potent tubulin inhibitors. We demonstrate that EAPB0203 and EAPB0503 are capable of blocking human melanoma cells in G2 and M phases and inducing cell death and apoptosis. Second, we show that EAPB0203 and EAPB0503, but also unexpectedly imiquimod, bind directly to purified tubulin and inhibit tubulin polymerization. As suggested by molecular docking and binding competition studies, we identify the colchicine binding site on β-tubulin as the interaction pocket. Furthermore, we find that EAPB0203, EAPB0503 and imiquimod display antagonistic cytotoxic effect when combined with colchicine, and disrupt tubulin network in human melanoma cells. We conclude that EAPB0203, EAPB0503, as well as imiquimod, interact with tubulin through the colchicine binding site, and that the cytotoxic activity of EAPB0203, EAPB0503 and imiquimod is correlated to their tubulin inhibiting effect. These compounds appear as interesting anticancer drug candidates as suggested by their activity and mechanism of action, and deserve further investigation for their use in the clinic.

## Introduction

Imiquimod (Aldara®) is a commercially available drug approved by the US Food and Drug Administration in 1997 to treat actinic keratosis, external genital warts, and superficial basal cell carcinoma [1]. Imiquimod is also under evaluation and/or currently used off-label in various malignancies. Efficacy against melanoma was demonstrated in a mouse model [2]. Used alone, imiquimod was able to clear an invasive melanoma in a 93-year-old woman [3]. In recent clinical trials, imiquimod used in combination was also proved efficient to treat superficial cutaneous melanoma metastases [4–6]. However, imiquimod is approved only as a topical cream, because it induced significant side effects that led to dose reduction or cure stop when given orally to cancer patients in a phase I clinical trial [7]. Even used as a topical treatment, imiquimod induces uncommon systemic side effects [8]. This underlines the usefulness of developing analogues with better efficiency and/or less general toxicity.

**Fig 1:**
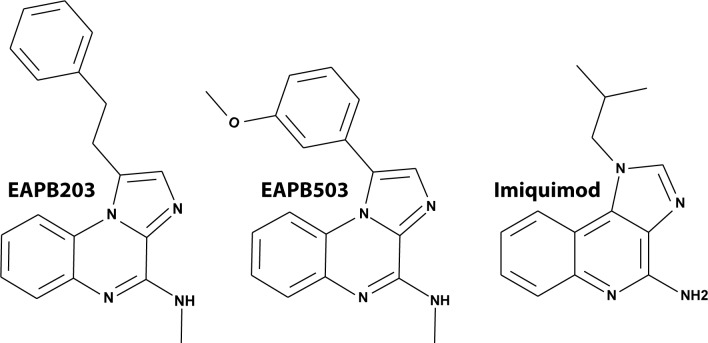
Chemical structures of studied compounds. The imidazo[1,2-*a*]quinoxalines family, nick-named *imiqualines*, was developed from structural analogy with imiquimod. The compound EAPB0203 (*N*-methyl-1-(2-phenylethyl)imidazo[1,2-a]quinoxalin-4-amine) was identified as the initial leader of the series. Further pharmacomodulation of imiqualines lead to the new compound EAPB0503 (1-(3-methoxyphenyl)-*N*-methylimidazo[1,2-*a*]quinoxalin-4-amine).

A series of heterocyclic compounds, the imidazo[1,2-*a*]quinoxalines family, nick-named *imiqualines*, was developed from structural analogy with imiquimod and synthesized by C. Deleuze-Masquefa and P.-A. Bonnet group [9,10]. These compounds displayed a direct antiproliferative effect on A375 highly resistant human melanoma cancer cell line, and the compound EAPB0203 (*N*-methyl-1-(2-phenylethyl)imidazo[1,2-a]quinoxalin-4-amine) was identified as the initial leader of the series [11] (Fig 1). The 50% inhibitory of maximum concentration (IC_50_) obtained in the A375 model for EAPB0203 was 1.57 μM, which was far lower than that obtained with imiquimod (70.3 μM) [11]. EAPB0203 was also cytotoxic at μM concentrations in adult T-cell leukemia/lymphoma (ATLL) cell lines and primary cells [12], and in chronic myeloid leukemia (CML) cell lines [13]. Interestingly, while imiquimod induced dose-limiting lymphocytopenia in phase I clinical trials [7,14], EAPB0203 10 μM was not cytotoxic toward non-malignant T lymphocytes. EAPB0203 also significantly inhibited tumor growth in nude mice xenografted with M4Be human melanoma cell line, to a greater extent than fotemustine [11], which was the second most commonly used first-line systemic treatment for metastatic melanoma in Europe [15].

Further pharmacomodulation of imiqualines lead to new compounds [16]. Among them, EAPB0503 (1-(3-methoxyphenyl)-*N*-methylimidazo[1,2-*a*]quinoxalin-4-amine) displayed a roughly ten times stronger cytotoxic activity than EAPB0203 in A375 cells (IC_50_ = 0.2 μM) [16] and in CML cells [13]. These results were confirmed by the NCI-60 DTP Human Tumor Cell Line Screen (http://dtp.cancer.gov/), which showed a mean GI_50_ (50% Growth Inhibitory Concentration) of 1.12 μM for EAPB0203 and of 0.490 μM for EAPB0503 in the 60 human tumor cell lines tested.

Interestingly, sub-acute toxicity studies in Sprague Dawley rats receiving intravenous EAPB0203 at 5 mg/kg once daily or EAPB0503 at 3 mg/kg for five consecutive days showed no effect on vital organs nor on blood components [17]. Pharmacokinetic properties and metabolism of EAPB0203 and EAPB0503 have been extensively studied. Metabolism occurred through demethylation and hydroxylation reactions involving mainly cytochrome P450 3A [18].

Therefore, understanding the mechanism of action of these promising therapeutic anticancer molecules, displaying strong cytotoxic activity and low toxicity, is an important step to assess their value for further development. Interestingly, TLR 7/8 agonism exerted by imiquimod is considered the main mechanism explaining its anticancer activity on melanoma, via the induction of pro-inflammatory cytokines leading to activation of DC cells/innate immunity and thereby Th1 antitumoral cellular immune response along with the activation of NF-KB [19]. Independently of TLR-7 and TLR-8 pathways, imiquimod has also been shown to be involved in adenosine signaling via receptor-independent adenylyl cyclase inhibition, the activation of NF-KB, and has also been shown to induce apoptosis of tumor cells at higher concentrations through secondary molecular mechanisms that have not been clearly elucidated. This pro-apoptotic activity involves caspase activation dependent of Bcl-2 [20]. Interestingly, anti-microtubule agents are now known to induce apoptosis via inhibition of anti-apoptotic Bcl-2 family proteins [21]. Unlike imiquimod which displays pro-inflammatory properties [22], imidazoquinoxalines showed an anti-inflammatory activity [23] associated to TNF-alpha production impairment through activation of p38MAPK pathway and inhibition of PI3K pathway in L929 murine fibroblast cell line. EAPB0203 has been shown to impair cell growth, block cell cycle in G2/M phase, and activate the mitochondrial pathway leading to apoptosis in ATLL cells. EAPB0203 negatively regulated anti-apoptotic proteins like c-IAP-1 and Bcl-XL, induced a loss of mitochondrial membrane potential, cytochrome c cytoplasmic release, and caspases 3 and 9 activation in malignant T cells. EAPB0203 also stabilized the pro-apoptotic proteins p53 and p21 in a dose- and time-dependent manner, activated p38MAPK pathway, and inhibited PI3K pathway [12]. More recently, EAPB0503 and EAPB0203 have been shown to inhibit AR320, K562 and LAMA84 CML cell lines growth. EAPB0503 induced cell cycle arrest in M phase and apoptosis, and down-regulated BCR-ABL protein. Cell growth inhibition was synergistic with imatinib, and imatinib-resistant cells were sensitive to EAPB0503 [13].

The COMPARE analysis [24] of NCI 60 Cell Line screening assay results for EAPB0203 and EAPB0503 *versus* the standards list available at NCI showed high similarity to antimicrotubule agents, particularly maytansine. Based on this information, EAPB0503 and other newly synthetized derivatives of the imidazoquinoxaline family have recently been shown to inhibit tubulin polymerization [25]. The aim of the present study was thus to evaluate EAPB0203 and EAPB0503 interaction with tubulin, in comparison with imiquimod.

## Materials and methods

### Cell culture

Melanoma A375 cell line was kindly provided by the cell culture facility of IRCM (Institut de Recherche en Cancérologie de Montpellier, France). Cell culture products were obtained from Lonza (Levallois, France). Culture medium was RPMI 1640, supplemented with 10% heat-inactivated (56°C) fetal bovine serum, 1% penicillin-streptomycine 5000 U/mL, and 1% L-glutamine 200 mM. Cells were maintained in a humidified atmosphere of 5% CO_2_ at 37°C. Cells were subcultured as to be maintained in the exponentially growing state, cell confluence never exceeding 90%. Trypsin-versene (EDTA) was used to detach the cells, and Dulbecco’s Phosphate Buffered Saline (DPBS) for washes.

### Compounds and reactants

EAPB0203 and EAPB0503 were synthesized as previously described [9,13,16]. Compounds and reactants were bought from Sigma-Aldrich (Saint-Quentin Fallavier, France) unless otherwise stated. Imiquimod was obtained from Molekula (Wessex House, Shaftesbury, Dorset, UK). EAPB0203, EAPB0503, imiquimod, colchicine, vinorelbine, nocodazole and warfarin were prepared as 0.1 M stock solutions in dimethyl sulfoxide (DMSO), and stored at -80°C until use. Working solutions of 0.1 or 1 mM were freshly prepared in culture medium for cell experiments, or in appropriate buffer (see below) for purified tubulin experiments. Final concentration of DMSO never exceeded 0.1% in cell culture medium.

### Proliferation kinetics

A375 cells were plated in 6-well plates at 600,000 cells/well density. Cells were treated 24 hours later with two concentrations of EAPB0203 (0.5 and 5 μM) and of EAPB0503 (0.05 and 0.5 μM) bounding their respective IC_50_. Stock solutions were diluted in culture medium to obtain the desired concentrations. Control wells received fresh culture medium alone. Time of treatment was considered time zero. At each time point, supernatant was withdrawn and cells were harvested by trypsinization. Supernatant and cell suspension were diluted together in culture medium, then centrifuged for 5 min at 1400 rpm to remove trypsin. Cells were resuspended in 500 μL DPBS, then counted using CASY Cell Counter (Roche Diagnostics, Meylan, France). In parallel, 100 μL of cell suspension were mixed with 25 μL Trypan Blue solution 0.4% for dead cells staining, and percentage of dead cells was determined by counting at least 200 cells in various fields using a Malassez counting cell. Dead cells were removed from the total cell count to obtain the number of living cells per well.

### Cell cycle: Staining of cells in G2 and M phase

A375 cells plating, treatment and harvest were the same as described for proliferation kinetics, and were performed in parallel. Nocodazole 50 ng/mL was used as a positive control of mitosis phase blockade [26]. Cells were harvested 24 hours after treatment (16 hours for Nocodazole). After centrifugation, cells were washed by dilution in DPBS containing BSA 0.5%, then centrifuged again. Cells were washed twice again in DPBS alone. The final pellet was resuspended in 100 μL DPBS, and 0.9 mL methanol at -20°C was added drop by drop for fixation. The mixture was kept on ice for 30 minutes, then frozen at -20°C. Prior to flow cytometry, 3 mL DPBS-BSA 0.5% were added, and cells centrifuged again. Cell pellet was resuspended in 100 μL DPBS-BSA 0.5%. After a 30 minute-incubation at room temperature, 2 μL of anti-Phospho-Histone H3 (Ser10) (D2C8) XP® Rabbit monoclonal antibody (phycoerythrin conjugate) (anti-PH3) (Cell Signaling Technology, Ozyme, Saint-Quentin en Yvelines, France) were added. A propidium iodide (PI)-RNase solution was prepared by diluting PI solution 1 mg/mL in DPBS to 1.6 μg/mL, and adding 0.1 mg RNase per mL. After 1 hour incubation at room temperature, cells were washed twice with 1 mL DPBS, then resuspended in 500 μL PI-RNase solution. Flow cytometry analysis was performed with FACS Calibur 2 (Plateforme Montpellier RIO Imaging, France), using two fluorescence channels. Cells in G2/M (stained with PI) and in M (stained with PI and anti-PH3) phases were quantitated using FlowJo software.

### Cell death and apoptosis

A375 cells plating, treatment and harvest were the same as described for proliferation kinetics. Cells were harvested 24, 48 and 72 hours after treatment. Treated cells were double-stained using Annexin V-FITC /7-AAD kit (Beckman Coulter, Villepinte, France), following the provider’s procedure. Briefly, binding buffer was diluted to tenth with distilled water and kept on ice. Harvested cells were washed with DPBS-BSA 0.5%, then with DPBS and finally with 1 mL ice-cold DPBS, then centrifuged at 4°C. Cell pellets were resuspended in 200 μL 1X binding buffer and kept on ice. 10 μL Annexin V-FITC solution and 20 μL of 7-AAD Viability Dye were added to 100 μL cell suspension. After a 30-minute incubation on ice in the dark, 400 μL 1X binding buffer were added. Samples were kept on ice until flow cytometry analysis, within a time period not exceeding 30 minutes. Flow cytometry analysis and quantitation of dead cells (Annexin V and 7-AAD positive) and apoptotic cells (Annexin V positive and 7-AAD negative) were performed as described above for cell cycle.

### Tubulin binding evaluation by Surface Plasmon Resonance (SPR)

Tubulin was prepared from pig brain according to the purification procedure described by Williams and Lee [27]. Surface Plasmon Resonance (SPR) technology was used to evaluate binding to purified tubulin. All analyses were performed on T200 apparatus (GE Healthcare, Montpellier, France) at 25°C. Purified tubuline was covalently immobilized on a CM5 flow cell sensor chip (GE Healthcare) by EDC/NHS (N-(3-dimethylaminopropyl)-N’-ethylcarbodiimide hydrochloride / N-hydroxysuccinimide) activation according to the manufacturer’s instructions. Reference flow cells were prepared with the same activation procedure, but without protein or with an irrelevant protein (Anti-GST antibody). EAPB0203, EAPB0503 and imiquimod were injected at different concentrations in HBS-EP+ buffer adjusted at 3% DMSO at a flow rate of 30 μL/min on the different flow cells. Concentrations tested were limited by aggregation phenomenon, which occurred for EAPB0203 above 25 μM, as determined by dynamic light scattering, with an increased particle size and a polydispersity index superior to 0.7 (S1 Fig). No aggregation was observed for colchicine at 200 μM. The binding values were collected after subtraction of the reference flow cell response and solvent correction. Each experiment series included blanks (running buffer), colchicine and warfarin as positive and negative control respectively. The standard error was calculated from four different experiments.

### Polymerization of purified tubulin *in vitro*

Tubulin was prepared from pig brain according to the purification procedure described by Williams and Lee [27]. Tubulin polymerization was monitored turbidimetrically at 350 nm with a MC2 spectrophotometer (Safas, Monaco) equipped with a thermal-jacketed cuvette holder. The reaction mixture was prepared at 0°C, and contained PEM buffer, 25% glycerol (v/v), 1 mM Guanosine Tri Phosphate (GTP), and 2.4 μM tubulin. GTP and tubulin were added at the very last minute. EAPB0203, EAPB0503, imiquimod and colchicine stock solutions were diluted in DMSO to the desired concentration, and 1 μL of the compound solution was added to the reaction medium. The same volume of DMSO alone was used for negative control. For testing of high concentrations of imiquimod, and for warfarin used as negative control, 20 μL of diluted imiquimod, warfarin or of DMSO alone had to be added. The final volume of the sample was 200 μL. The reaction was started by placing the cuvette in the spectrophotometer cell compartment thermostated at 37°C. Ice was added 45 minutes later to initiate depolymerization to check for signal specificity.

Kinetics of purified tubulin assembly *in vitro* was characterized by parameters A_max_, t_1/10_, p and k_obs_, as described by Bonfils *et al* [28]. A_max_ is the maximum absorbance plateau value measured in the assay, and reflects polymerized tubulin amount. t_1/10_ is the abscissa value of the A_max_/10 absorbance. p is the slope of the plot log(A(t)/A_max_) *versus* log(t) during the elongation process, *ie* from 1 min to the time of 80% A_max_. The value of p is indicative of the number of successive steps in the nucleation process. The pseudo-first order rate constant of elongation, k_obs_, is determined by plotting ln(1 –A(t)/ A_max_) as a function of time.

### Microtubule network observation by immunofluorescence

A375 cells plating and treatment were the same as described for proliferation kinetics. A375 cell line was treated for 24h with EAPB0203, EAPB0503 and imiquimod at various concentrations (2 and 5 times their respective IC_50_ as determined in A375 cells). Colchicine (1 μM) was used as positive control. Culture medium with a similar DMSO concentration was used as negative control. Interphase microtubule network was visualized by direct immunofluorescence. Culture medium was removed and cells fixed with methanol. Cells were then incubated with a mouse monoclonal anti-β-tubuline antibody (Mouse clone TUB 2.1, T4026, Sigma-Aldrich) for 1.5h, followed by incubation with a secondary Rhodamine-labeled anti-mouse antibody for 1.5h. After washing, cells were colored with Hoechst (Life Technologies, Saint-Aubin, France). Microtubule network (green) and nuclear DNA (red) were visualized using a Leica DMRM fluorescence microscope with a 63x magnification. Images were obtained using a JAI CV-M1 camera and Isis software, and merged using Adobe photoshop software.

### Combination with colchicine and vinorelbine for cytotoxicity

A375 cells were seeded in 96 well-plates at 20,000 cells per well. Treatment was applied 24 hours later. Control cells received culture medium only. Higher concentrations for the test were chosen based on previously determined IC_50_ values, and were 2.81 x 10^-5^M for EAPB0203, 1.6 x 10^-6^M for EAPB0503, 1.34 x 10^-7^M for colchicine, 3.06 x 10^-4^M for vinorelbine, and 1.17 x 10^-4^M for imiquimod (for IC_50_ evaluation method, see [16]). For each combination test, cells were treated with each compound alone and with the constant ratio combination, at the higher concentrations chosen and concentrations obtained by nine serial half dilutions. Cell viability was assessed 48h after treatment, using the 3-(4,5-Dimethyl-2-thiazolyl)-2,5-diphenyl-2H-tetrazolium bromide (MTT) test. Chou and Talalay method was used to calculate a combination index [29,30], using the CalcuSyn software (Biosoft, Cambridge, UK). Briefly, the median effect equation correlates the drug dose to cytotoxicity as follows:

$$fa/fu={\left(D/Dm\right)}^{m}$$

where D is the dose, Dm is the dose necessary to obtain the median effect, fa is the fraction affected by the dose and fu is the fraction unaffected by the dose (fu = 1 –fa), and m embodies the sigmoidicity of the dose-effect curve. This equation is used to calculate Dx, the dose that kills x % of cells. The Combination Index (CI) is then calculated as follows:

$$CI={\left(D\right)}_{1}/{\left(Dx\right)}_{1}+{\left(D\right)}_{2}/{\left(Dx\right)}_{2}$$

CI<1, CI = 1 and CI>1 respectively mean a synergy, an additive effect or an antagonism.

### Molecular modeling of interaction with tubulin

Molecular docking *in silico* was used to investigate the possible binding modes of EAPB0203, EAPB0503 and imiquimod on the colchicine binding site of tubulin. The AutoDock Vina program was used [31]. AutoDock Tools [32] was used for tubulin (PDB1SA0, *Bos taurus*) and ligands preparation to generate pdbqt files. Water molecules and colchicine were removed from PDB1SA0, polar hydrogens and Gasteiger partial charges were added. Ligands were obtained on the data base ZINC [33] in.mol2 files, and prepared the same way, with assignment of all flexible covalent bonds. A grid of 40x40x40 points in x,y,z axes and a space of 0.375 Å was centered on colchicine binding site (center: 116, 556x89, 199x6,541). For each run, the first ten more stable conformations were considered. Docking results were handled using the opensource PyMOL Molecular Graphics System, Version 1.7.4 Schrödinger, LLC. Predicted binding affinity is calculated based on the score function used by Autodock Vina (ΔG = ΔG_H-bond_ + ΔG_vdw_ + ΔG_hydrophobic_ + ΔG_comformation_), which is composed of a conformation-dependent component (including intra- and intermolecular steric, hydrophobic and hydrogen interactions) and a conformation-independent component (taking into account the number of rotative bonds between ligands atoms). Each contribution is given a different weight to score function [34].

### Competition with colchicine for tubulin binding: Fluorescence of the colchicine-tubulin complex

We used an intrinsic property of colchicine, which makes a fluorescent complex when bound to tubulin (λ_exc_ = 365nm, λ_em_ = 435nm) but is not fluorescent in solution [35]. Tubulin (3 μM) was incubated with colchicine (3 μM) for 30 minutes at 37°C to form complexes. EAPB0203, EAPB0503, imiquimod, nocodazole (positive control) and vinorelbine (negative control) were then added at different concentrations (5, 15, 20 and 30 μM), then incubated for 60 minutes at 37°C. Fluorescence spectra were recorded after excitation at 365 nm. Fluorescence was corrected for blank and imiqualines slight fluorescence when necessary with equimolar solutions.

### Data analysis

Linear regressions and statistical tests were performed using GraphPad Prism software (GraphPad Software, Inc., La Jolla, USA).

## Results

### EAPB0203 and EAPB0503 inhibit cell proliferation and display cytotoxicity

**Fig 2:**
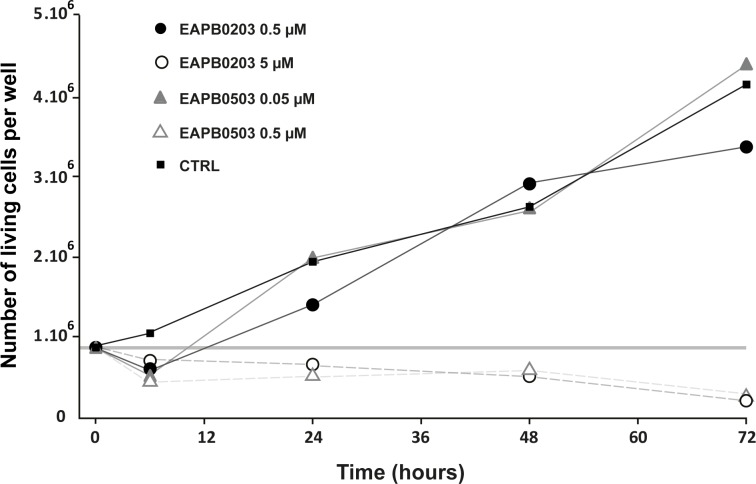
EAPB0203 and EAPB0503 inhibit cell proliferation and display cytotoxicity. A375 cells were treated with EAPB0203 or EAPB0503 at the indicated concentrations, harvested at various times post-treatment, and counted using Casy cell counter. Percentage of dead cells was determined on Malassez hemocytometer after trypan blue staining, and dead cells removed from the total cell count. The thick grey line corresponds to the number of living cells as counted at time zero.

EAPB0203 and EAPB0503 at the highest concentration tested (5 and 0.5 μM respectively) totally inhibited A375 cell growth until 72h (Fig 2), the number of cells decreasing slightly with time. When used at the lowest concentration (0.5 and 0.05 μM respectively), EAPB0203 and EAPB0503 had no effect on cell proliferation kinetics. Interestingly, EAPB0503 at 0.5 μM concentration had a similar effect as EAPB0203 at 5 μM, confirming its roughly ten times higher cytotoxic activity.

### EAPB0203 and EAPB0503 block cell cycle in G2 and M phases and induce apoptosis

**Fig 3:**
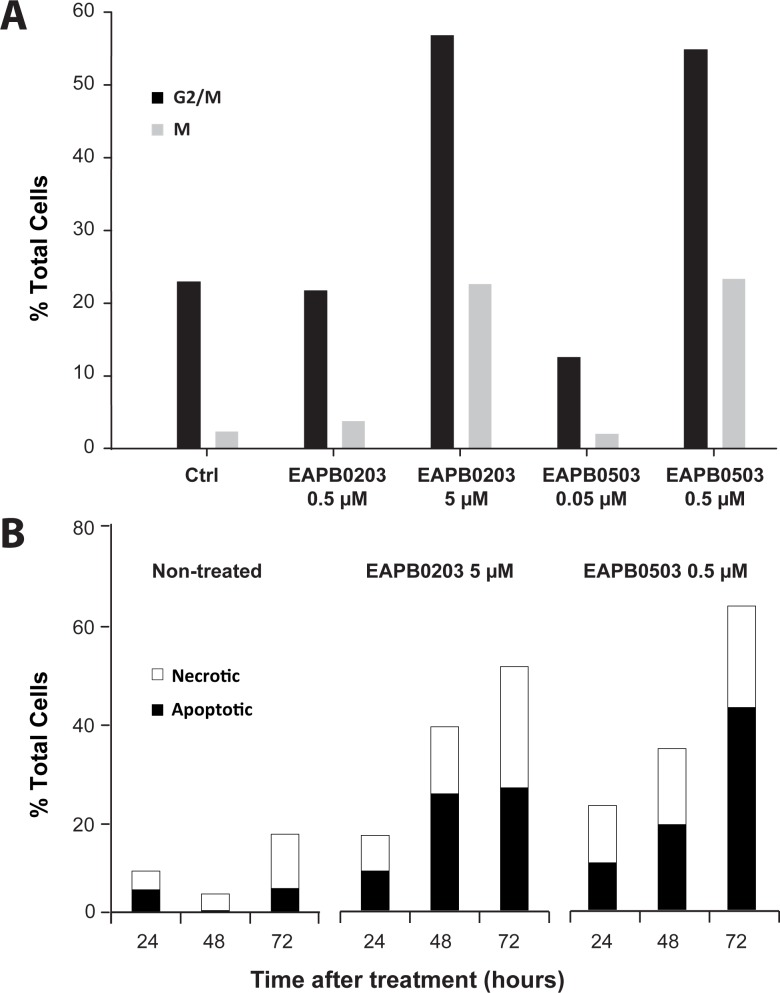
EAPB0203 and EAPB0503 block cell cycle in G2 and M phases and induce apoptosis. A375 cells were treated with EAPB0203 or EAPB0503 at the indicated concentrations. (**A**) After 24 hours, cells were stained with propidium iodide alone (G2/M) or with propidium iodide and anti-phospho histone H3 antibody (M). Flow cytometry was used to determine the percentage of cells in G2/M and M phases of the cell cycle. (**B)** After the indicated time, cells were stained with 7-AAD and Annexin V. We used flow cytometry to determine the percentage of necrotic and secondary necrotic cells (Annexin V and 7-AAD positive) and early stage apoptotic cells (Annexin V positive and 7-AAD negative).

Nocodazole 50 ng/mL used as positive control led to 37.8% (Standard Deviation (SD) 9.87) cells blocked in M phase after 16h treatment. EAPB0203 and EAPB0503, respectively at 5 and 0.5 μM, induced accumulation of treated A375 cells in G2/M and M phase after 24h (Fig 3). Proportion of cells in G2/M grew from 23.0% (negative control) to 56.9% (EAPB0203 5 μM) and 54.9% (EAPB0503 0.5 μM). Consistently, proportion of cells in M grew from 2.35% to 22.7% and 23.4% respectively. Accumulation in M was not sufficient to account for G2/M accumulation, suggesting also G2 accumulation. Increase in mitotic index was associated with a decrease in cells in G0/G1 (first Peak), as illustrated in S2 Fig. Accumulation of treated cells in G2/M and M phases was negligible at 48h, and totally disappeared at 72h. EAPB0203 5 μM induced apoptosis in more than 20% of the cells after 48 and 72h, while percent of apoptotic cells remained below 5% for untreated cells (Fig 3). Percent of necrotic cells strikingly increased from 13.8% after 48h to 25% after 72h, while the maximum percent of dead cells for untreated cells was 11.2% after 72h. A similar trend was observed for EAPB0503 0.5 μM, but the percent of apoptotic cells reached 41.6% after 72h. Representative cytometry results are displayed in S3 Fig.

### EAPB0203, EAPB0503 and imiquimod bind tubulin

**Fig 4:**
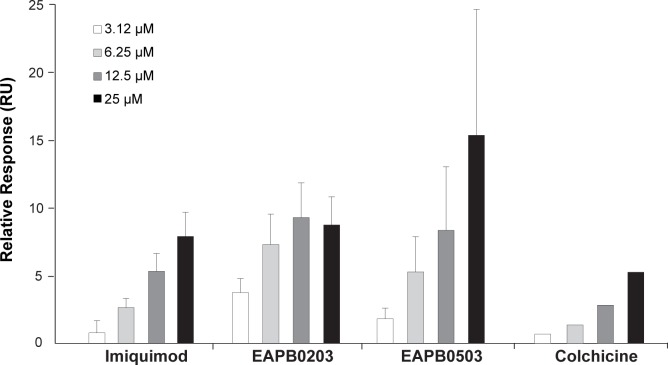
EAPB0203, EAPB0503 and imiquimod bind tubulin. Binding levels of EAPB0203, EAPB0503 and imiquimod were determined by surface plasmon resonance on immobilized tubulin at different concentrations (n = 4, except for colchicine n = 1).

A SPR approach was used to evaluate the direct binding of EAPB0203, EAPB0503 and imiquimod to tubulin immobilized on a CM5 sensor chip. The experiment was validated by a dose effect experiment performed on colchicine (S4 Fig). The resulting K_D_ of 21 μM was in accordance with the literature [36]. As expected, warfarin showed no binding to tubulin with no detectable signal. A specific dose-dependent binding to tubulin was observed for EAPB0203, EAPB0503 and imiquimod (Fig 4). A linear regression was performed between test compound concentrations and binding response. Slope was significantly different from zero for imiquimod, and EAPB0503, but not EAPB0203. The binding responses of test compounds were higher than colchicine responses (Fig 4), but under or equal to the calculated stoichiometric binding level (20–25 RU). K_D_ could not be determined due to higher concentration limit associated to aggregation.

### EAPB0203, EAPB0503 and imiquimod inhibit purified tubulin polymerization

**Fig 5:**
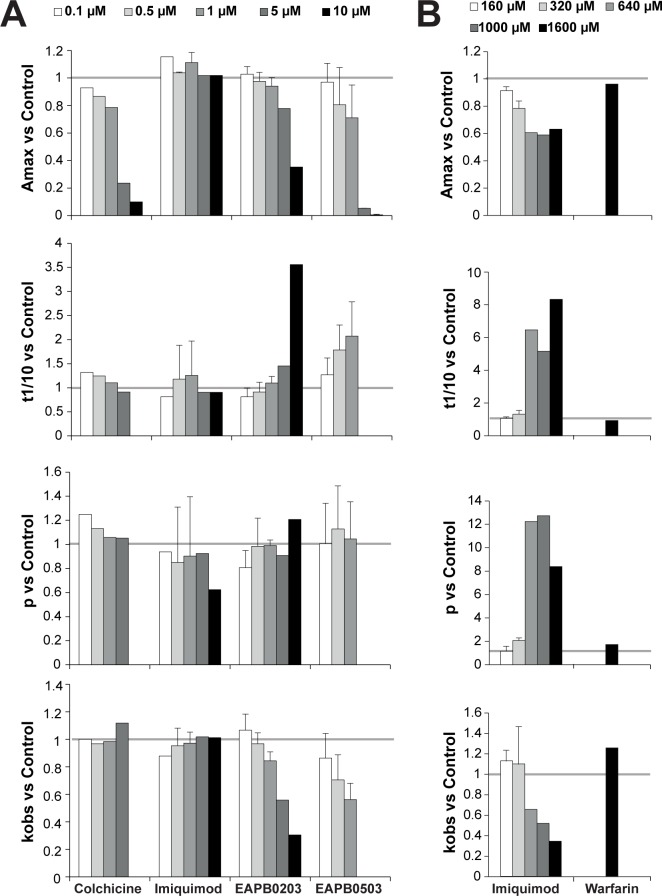
EAPB0203 and EAPB0503, and imiquimod at high concentrations, inhibit polymerization of tubulin *in vitro*. Purified tubulin polymerization was quantitated by turbidimetry measurement at 350 nm with or without (blank) various concentrations of EAPB0203, EAPB0503, imiquimod, and colchicine as positive control, or warfarin (1,600 μM) as negative control. Various parameters representative of the polymerization process were calculated: A_max_ (maximum absorbance plateau value), t_1/10_ (abscissa value of the A_max_/10 absorbance), p (slope of the plot log(A(t)/A_max_) versus log(t) during the elongation process, i.e. from 1 min to the time of 80% A_max_), and k_obs_ (pseudo-first order rate constant of elongation, determined by plotting ln(1 –A(t)/ A_max_) as a function of time). The ratio to blank values are reported here. The red line embodies the ratio of 1, meaning identity to blank. Mean ± SD, n = 1 to 6. (**A)** EAPB0203, EAPB0503, imiquimod and colchicine at 0.1 to 10 μM concentrations. (**B)** Imiquimod at 160 to 1,600 μM concentrations. Mean ± SD, n = 1 or 2.

As shown in Fig 5, at concentrations lower than or equal to 10 μM, EAPB0203 and EAPB0503, as well as colchicine, dose-dependently inhibited polymerization of tubulin, while imiquimod had no effect. Indeed, the parameter A_max_, considered as proportionally related to the mass concentration of tubulin polymer [28], was decreased by EAPB0203, EAPB0503 and colchicine. Strength of the effect was differential, with EAPB0503 > colchicine > EAPB0203. Polymerization was totally prevented by colchicine at 10 μM, and EAPB0503 at 5 and 10 μM. Nucleation process seemed to be delayed by EAPB0203 and EAPB0503, but not colchicine, as reflected by the increase in t_1/10_, while no effect was observed on p parameter. The pseudo-first order elongation rate k_obs_ was decreased by EAPB0203 and EAPB0503, but not colchicine, suggesting a slowdown and impairment of elongation process. Since imiquimod IC_50_ in A375 cells (70.3 μM) was much higher than IC_50_ of EAPB0203 or EAPB0503 (1.57 and 0.2 μM respectively) [11], we assessed the effect of imiquimod on tubulin polymerization at higher imiquimod concentrations. From 320 μM, imiquimod decreased A_max_ and k_obs_, and increased t_1/10_ and p (Fig 5). Hence, imiquimod’s effect on tubulin polymerization displayed the same profile as EAPB0203 and EAPB0503, except for the increase in p, suggestive of an increase of nucleus size. A linear regression was performed between test compound concentrations and each polymerization parameter, which confirmed our observations. Indeed, for A_max_, t1/10 and k_obs_, slope was significantly different from zero for imiquimod, EAPB0203 and EAPB0503. For the parameter “p”, slope was significantly different from zero for imiquimod only.

### EAPB0203, EAPB0503 and imiquimod disturb microtubule network in A375 cells

**Fig 6:**
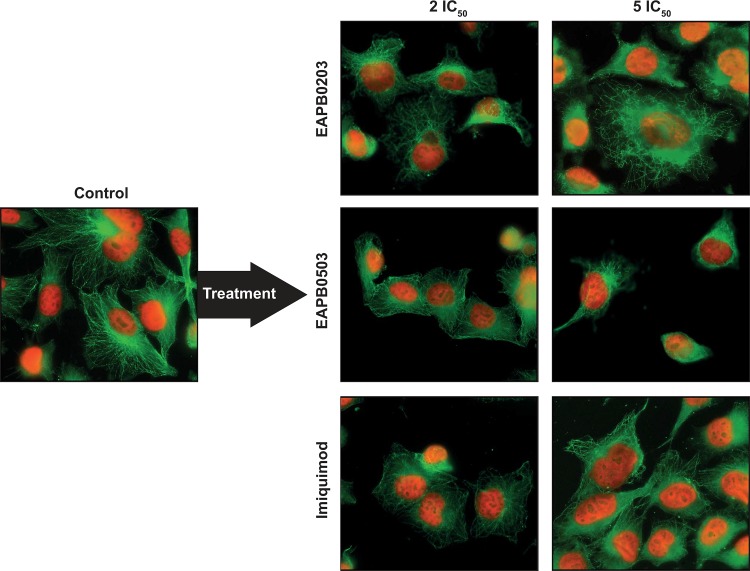
EAPB0203, EAPB0503 and imiquimod disturb microtubule network in A375 cancer cell line. A375 cells were treated by EAPB0203, EAPB0503 and imiquimod at the indicated concentrations for 24h. Beta-tubulin was stained using a mouse monoclonal anti-β-tubulin antibody and a secondary Rhodamine-labeled anti-mouse antibody. Nuclei were stained with Hoechst. Microtubule network (green) and nuclear DNA (red) were visualized using a Leica DMRM fluorescence microscope with a 63x magnification. Representative images are displayed here.

A homogeneous well-defined microtubule network was observed for control cells, with microtubules orientated from the center to the periphery of the cell. Many mitotic cells with a mitotic spindle were visualized. EAPB0203, EAPB0503 and imiquimod markedly disorganized the microtubule network at a concentration of 5 times their respective IC_50_ (Fig 6), in a manner that differed from colchicine, which totally prevented microtubule polymerization (S5 Fig). The cytoskeleton was inhomogeneously distributed, morphology was rounder, and adhesion to coverslips was reduced, which might be associated to destabilization of tubulin cytoskeleton. Later than 24h, very few mitotic cells could be observed, consistently with apoptotic death of cells blocked in M as evaluated by flow cytometry. These qualitative observations illustrate that, in addition to interacting with purified tubulin *in vitro*, the test compounds also modify microtubule structure in cells.

### EAPB0203, EAPB0503 and imiquimod display an antagonist cytotoxic effect with colchicine

**Fig 7:**
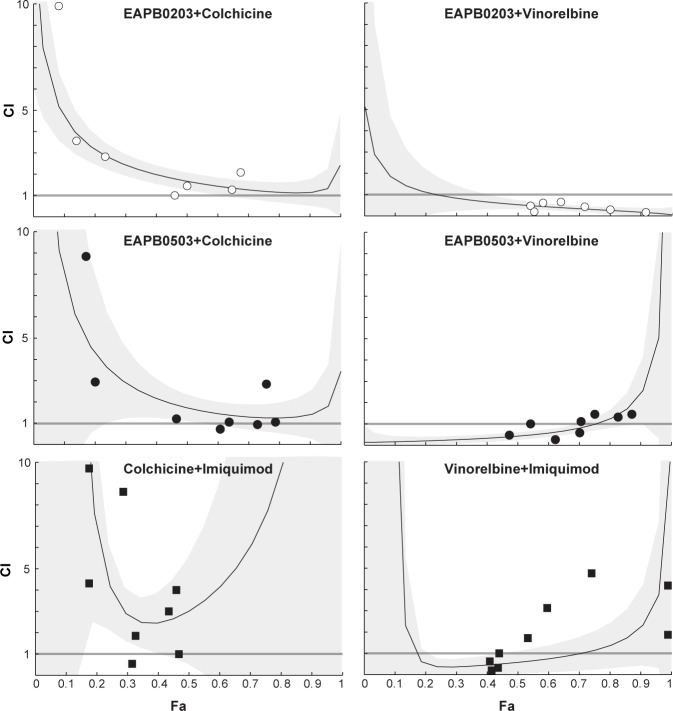
EAPB0203, EAPB0503 and imiquimod display an antagonistic effect with colchicine, but synergistic with vinorelbine. A375 cells were treated with EAPB0203, EAPB0503, colchicine and vinorelbine–alone and with constant ratio combination—at concentrations surrounding their previously determined IC_50_. Cell viability was assessed 48h after treatment, using the MTT test. Chou and Talalay method was used to calculate a combination index: CI<1, CI = 1 and CI>1 respectively mean a synergy, an additive effect or an antagonism. The grey frame embodies the 90% confidence interval as determined by Calcusyn software. Fa = fraction affected by the dose.

Most compounds that inhibit tubulin polymerization bind two major domains of tubulin: vinca and colchicine domains [37,38]. In order to identify whether EAPB0203 and EAPB0503 would bind one of those well-identified sites, we combined them with colchicine and vinorelbine and measured the effect of the combination on cytotoxicity in A375 cells. Chou-Talalay method was used to calculate a combination index (CI) with Calcusyn software [29,30]. After calculation of CI at various concentrations (Fig 7), we found that EAPB0203, EAPB0503 and imiquimod were acting antagonistically to colchicine on cytotoxicity (CI>1). A synergistic effect was oppositely obtained with vinorelbine (CI<1), which was less clear for imiquimod. Consistently, a synergistic effect has been described for vinorelbine and colchicine combination [39]. These results strongly suggest that EAPB0203, EAPB0503 and imiquimod might bind tubulin on the colchicine binding site, and not on the vinca-alcaloïds binding site.

### EAPB0203, EAPB0503 and imiquimod can bind tubulin on the colchicine-binding site

**Fig 8:**
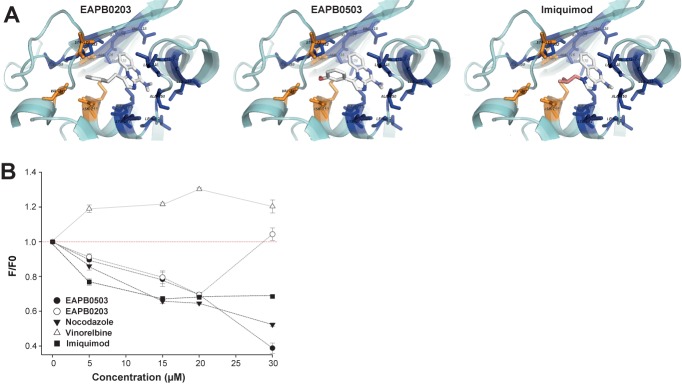
EAPB0203, EAPB0503 and imiquimod bind tubulin on the colchicine binding site. **(A)** Interaction modes of EAPB0203, EAPB0503 and imiquimod on the colchicine site as predicted by AutoDock Vina. Molecules interact with two hydrophobic pockets (blue and orange). (**B)** Competition of EAPB0203, EAPB0503, imiquimod, nocodazole (positive control) and vinorelbine (negative control) with colchicine for tubulin binding. Fluorescence of the colchicine-tubulin complex was measured after excitation at 365 nm. F/F0: relative fluorescence intensity, F0: fluorescence intensity of the colchicine-tubulin complex alone.

To confirm the hypothesis raised by SPR and combination studies, we first investigated the possible binding modes of EAPB0203, EAPB0503 and imiquimod on the colchicine binding site of beta-tubulin, using molecular docking *in silico*. The docking method was validated by redocking colchicine on the colchicine binding site: the experimental crystallography conformation and the lowest energy conformation predicted by Autodock Vina were similar (RMSD of 0,568 Å between the two structures) (S6 Fig). Among the binding modes generated for EAPB0203, EAPB0503 and imiquimod, the lowest energy modes corresponded to a superposition with colchicine (Fig 8).

Twenty-nine residuals were identified as being involved in colchicine binding to beta-tubulin [40]. The C ring of colchicine interacts with a specific zone (labeled in orange in Fig 8) through Van Der Waals contacts with Valα181, Serα178, and Valβ315. Interestingly, the same hydrophobic interactions were involved for EAPB0503 and EAPB0203, and to a lower extent for imiquimod, which does not contain a phenyl ring. Colchicine ring A is buried in a second hydrophobic pocket (labeled in blue in Fig 8). The methoxy group in 3 position is involved in a hydrogen bond with the thiol group of Cysβ241. This conformation was also found for EAPB0203, EAPB0503 and imiquimod, whose quinoxaline and quinoline aromatic rings are in close contact with Cysβ241. A similar conformation is frequently found among colchicine site pharmacophores, and is considered essential to activity [41]. Moreover, the conformation obtained is compatible with hydrophobic contacts between methylamine group and Alaβ250 and Leuβ242, and with an hydrogen bond to Cysβ241 (3.4 Å), which could explain the higher biological activity of EAPB0203 and EAPB0503 when compared to other compounds of the imidazo[1,2-*a*]quinoxalines family with a different substitution [9]. In the case of colchicine, in addition to the hydrogen bond to Cysβ241, a carbonyl group on the C ring is involved in a hydrogen bond to Valα181. This highly energetic bond to Valα181 is also found for EAPB0503, whose lowest energy conformation shows a favorable position of methoxyphenyl (3.1 Å). Autodock Vina algorithm predicts similar affinity for colchicine (score of -9.1 kcal/mol), EAPB0203 and EAPB0503 (both -8.9 kcal/mol), while imiquimod displays less affinity (-7.3 kcal/mol).

To confirm the involvement of the colchicine binding site of beta-tubulin in the interaction of EAPB0203, EAPB0503 and imiquimod with tubulin, we studied the competition of EAPB0203, EAPB0503 and imiquimod with colchicine for tubulin binding. When nocodazole (used as positive control of colchicine site binding) but also EAPB0203, EAPB0503 and imiquimod were added to colchicine and tubulin, fluorescence decreased dose-dependently (Fig 8), suggesting that these molecules were competing with colchicine for binding to tubulin. Contrarily, vinorelbine, which binds to another site, did not decrease fluorescence, and actually increased fluorescence, suggesting a potential synergistic action. Addition of 30 μM EAPB0203 induced no reduction of fluorescence, which might be related to aggregation phenomena disturbing the fluorescent signal (S1 Fig). These results strongly suggest that EAPB0203, EAPB0503 and imiquimod bind tubulin on the colchicine site.

## Discussion

Anti-microtubule agents are known to target the tubulin cytoskeleton and suppress microtubule dynamics, which leads to aberrant mitotic spindle, cell cycle blockade in G2/M phases, and finally induces apoptotic cell death [38]. Consistently, through a cluster of complementary results, we demonstrated in the present paper that EAPB0203 and EAPB0503, strong antiproliferative agents from the imidazo[1,2-*a*]quinoxalines series, display an anti-microtubule activity. First, we observed that EAPB0203 and EAPB0503 impaired A375 cell proliferation, blocked cell cycle in G2 and M phases, and induced apoptotic cell death. Second, we showed that EAPB0203 and EAPB0503 dose-dependently inhibited purified tubulin polymerization in a cell-free system, with a strength similar to colchicine, and at cellular level demolished microtubule network organization in A375 cell line. Finally, we demonstrated that EAPB0203 and EAPB0503 bound tubulin and interacted with the colchicine binding site. SPR analysis showed a direct dose-dependent specific binding to tubulin. Our molecular docking data suggested an interaction with the colchicine-site of β-tubulin, which was confirmed by colchicine-site binding competition studies. The latter finding was in line with combination studies, which showed an antagonistic cytotoxic effect of EAPB0203 and EAPB0503 with colchicine, but a synergistic cytotoxic effect with vinorelbine.

The present findings are consistent with previously published results regarding the mode of action of EAPB0203 and EAPB0503. EAPB0503 has recently been shown to inhibit tubulin polymerization [25]. EAPB0203 and EAPB0503 have been shown to impair cell growth, block cell cycle in G2/M phase, and induce apoptosis in ATLL cells (EAPB0203) and CML cells (EAPB0503). Furthermore, as shown in malignant T cells, EAPB0203 negatively regulated c-IAP-1 and Bcl-XL anti-apoptotic proteins, stabilized p53 and p21 pro-apoptotic proteins, and activated p38MAPK pathway [12]. Similar downstream regulation of p53, p21, c-IAP, Bcl-XL and MAPK has been described for anti-microtubule agents [42,43]. These findings are in favor of a close cause consequence relationship between antimicrotubule activity and the cytotoxicity mechanisms observed with these compounds. We envision that our next studies could benefit from further identification of the effects that contribute the most to cytotoxicity through a concentration dependent comparative analysis of cellular effects, which could in turn guide pharmacomodulation and inform on the pharmacological potential of these compounds. Due to their role in mitosis and cell division, microtubules and their dynamics are a major target for anticancer drugs, cancer cells being more vulnerable due to a higher rate of division [44]. Based on the success of the chemically diverse class of anti-microtubule drugs (with various tubulin-binding sites), it has been argued that microtubules represent the best cancer target identified so far. It seems likely that drugs of this class will continue to be major chemotherapeutic agents, despite the development of more selective approaches [44,45]. More specifically, the colchicine-site has been described as one of the most important target for tubulin polymerization inhibitors. In the hope of developing novel useful drugs with favorable pharmacological profiles, a large number of molecules with structural diversity has been identified as interacting with the colchicine site of tubulin and some have been shown to exert an anticancer activity by leading to G2 arrest and apoptosis [46,47]. For example, the molecule BAL27862 is currently in phase I clinical trial [48]. To our knowledge, among described pharmacophores of tubulin colchicine domain [48,49], no structure is related to imidazoquinoxaline structure. EAPB0203 and EAPB0503 hence represent unique colchicine domain binding compounds. In spite of the recent discovery of many novel pharmacophores, increasing the library of available compounds could facilitate the identification of appropriate pharmacokinetic properties in order to obtain a highly potent, low toxicity anti-microtubule agent for the treatment of cancers.

A totally unexpected and nevertheless major result was also obtained in the present study: we happened to observe for the first time that the marketed drug imiquimod might bind to the colchicine-binding site of tubulin, and could accordingly inhibit tubulin polymerization, although at higher concentrations than EAPB0203 and EAPB0503. Imiquimod was not included in proliferation, cell cycle or apoptosis tests because it displays a very low cytotoxicity in A375 cells (IC_50_ 70.3 μM), which is consistent with the insubstantial induction of cell death by imiquimod alone reported by Weber *et al*. [50]. Imiquimod was initially included in tubulin binding SPR assays as a negative control, which led to the unexpected discovery that imiquimod actually also bound tubulin. Imiquimod is a marketed and well-known drug. The main mechanism of action of imiquimod is associated to immune stimulation involving alpha-interferon induction [51] through modulation of Toll-like receptor 7 (TLR7) [52]. A direct pro-apoptotic effect of imiquimod has also been described in various skin cancer cell types, including melanoma, and involved the mitochondrial pathway of apoptosis, as suggested by Bcl-2–dependent cytosolic translocation of cytochrome c [53,54]. Treatment with imiquimod was also reported to induce cell cycle arrest at the G2/M phase in TRAMP-C2 mouse prostate cancer cells, and apoptosis via the mitochondrial-dependent pathway [55]. Consistently with our results, the above studies were conducted at high concentrations of imiquimod, at least 5 μg/mL (i.e. 20.8 μM, imiquimod weighing 240.3 g/mol). Although not formally demonstrating a causative link between interaction with tubulin and cytotoxic activity, we observed that the concentrations needed to inhibit tubulin polymerization (0.5, 5 and 320 μM for EAPB0503, EAPB0203 and imiquimod respectively) were in the same range as the cytotoxic concentrations (0.2, 1.57 and 70.3 μM for EAPB0503, EAPB0203 and imiquimod respectively) for the three molecules evaluated. In contrast, and as could be expected, effect on tubulin polymerization was not correlated to TLR7 agonist activity, which was observed at a much lower concentration for imiquimod (from 1 μg/mL or 4.16 μM) while no TLR7 agonist activity was observed for EAPB0203 and EAPB0503 even at 100 μg/mL (above 300 μM) (S7 Fig). However, as stated by Narayan *et al*. [22], the exact mechanism of action of imiquimod is still largely undefined. We discovered that imiquimod bound to tubulin and was able to inhibit purified tubulin polymerization. Furthermore, our results are consistent with the binding of imiquimod to the colchicine site of tubulin. To our knowledge, no interaction between imiquimod and tubulin had been reported before. Even though further studies are needed to confirm this interaction, our results bring a new stone to the understanding of the mechanism of action of imiquimod.

## Supporting information

S1 Fig Aggregation of EAPB0203, EAPB0503, colchicine and imiquimod as evaluated using dynamic light scattering. We measured the mean particle size of EAPB0203, EAPB0503 and imiquimod at different concentrations in HBS-EP+ buffer which was used in the SPR experiments presented in Fig 4. After dissolving the compounds with sonication, the solutions were allowed to equilibrate for 2 hours. Experiments were limited by aggregation phenomenon, which occurred for EAPB0203 above 25 μM with an increased particle size and a polydispersity index superior to 0.7.
(EPS)

S2 Fig Representative dot plot of cells in G2/M and M phases measured by flow cytometry, used to elaborate Fig 3A. A375 cells were treated with using Phospho-Histone H3 (phycoerythrin conjugate, PE) and propidium iodide (PI) as described in Materials and methods. Flow cytometry analysis was performed with FACS Calibur 2, using two fluorescence channels. Cells in G2/M (stained with PI) and in M (stained with PI and anti-PH3) phases were then analyzed using FlowJo software.
(EPS)

S3 Fig Representative dot plot of dead and apoptotic cells measured by flow cytometry, used to elaborate Fig 3B. A375 cells were harvested 24, 48 and 72 hours after treatment and double-stained using Annexin V-FITC /7-AAD kit as described in Materials and methods. Flow cytometry analysis and quantitation of dead cells (Annexin V and 7-AAD positive) and apoptotic cells (Annexin V positive and 7-AAD negative) were performed using the FlowJo software.
(EPS)

S4 Fig Evaluation of the affinity of colchicine to tubulin as measured by surface plasmon resonance. Kinetic response profile (**A**), and maximum response plotted against concentration of Colchicine (**B**). This dose effect experiment performed on colchicine enabled us to calculate a resulting K_D_ of 21 μM, in accordance with the literature, which permitted to validate our experimental set up to measure the affinity of EAPB0203, EAPB0503 and imiquimod to tubulin.
(EPS)

S5 Fig Colchicine (1 μM) prevents microtubule polymerization in A375 cancer cell line after 24h. Beta-tubulin was stained using a mouse monoclonal anti-β-tubulin antibody and a secondary Rhodamine-labeled anti-mouse antibody. Nuclei were stained with Hoechst. Microtubule network (green) and nuclear DNA (red) were visualized using a Leica DMRM fluorescence microscope with a 63x magnification. Two representative images are displayed here.
(EPS)

S6 Fig **Comparison of natural crystallographic conformation** (**A**) and conformation predicted by molecular docking (**B**) of colchicine on the colchicine site of beta-tubulin (PDB: 1SA0) using Autodock Vina. (**C**) Chemical structure of Colchicine.
(EPS)

S7 Fig Evaluation of TLR7 agonist activity of EAPB0503, EPAB0203 and imiquimod, in comparison with the control TLR7/8 agonist R848 (resiquimod). We observed activation of human and murine TLR7 reporters in HEK2903 cells for imiquimod from 1 μg/mL or 4.16 μM, while no TLR7 agonist activity was observed for EAPB0203 and EAPB0503 even at 100 μg/mL (above 300 μM). (**A**) Dose response to human TLR7 on NF-kB reporter HEK293 (HEK-Blue™-hTLR7, Invivogen) (**B**) Dose response to murine TLR7 on NF-kB reporter HEK293 (HEK-Blue™-mTLR7, Invivogen).
(EPS)

S1 File Experimental raw data and images used to generate all figures. (ZIP)
