# Supplementary figures and images for: Imidazoquinoxaline anticancer derivatives and imiquimod interact with tubulin: Characterization of molecular microtubule inhibiting mechanisms in correlation with cytotoxicity
Source: PLoS One. 2017 Aug 10;12(8):e0182022. doi: 10.1371/journal.pone.0182022 (PMC5552358; doi:10.1371/journal.pone.0182022)

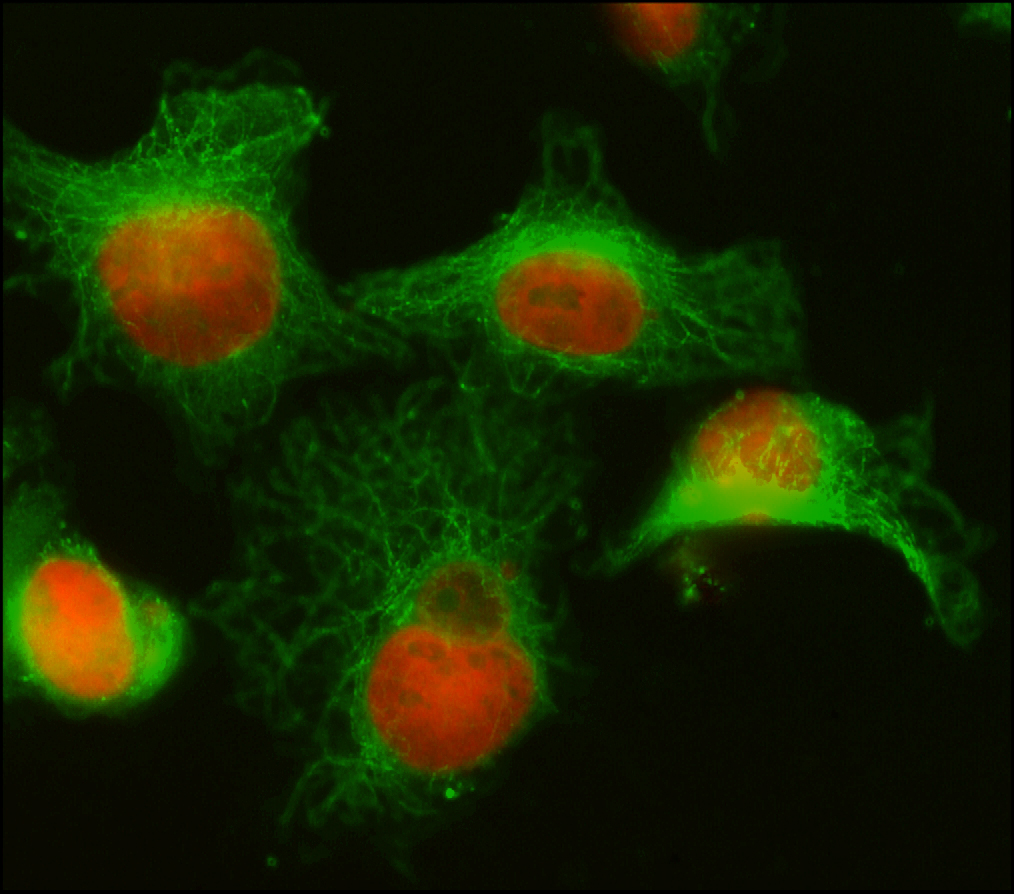

Supplement: S1 File — (ZIP) [file pone.0182022.s008.zip › 203_2IC50.jpg]

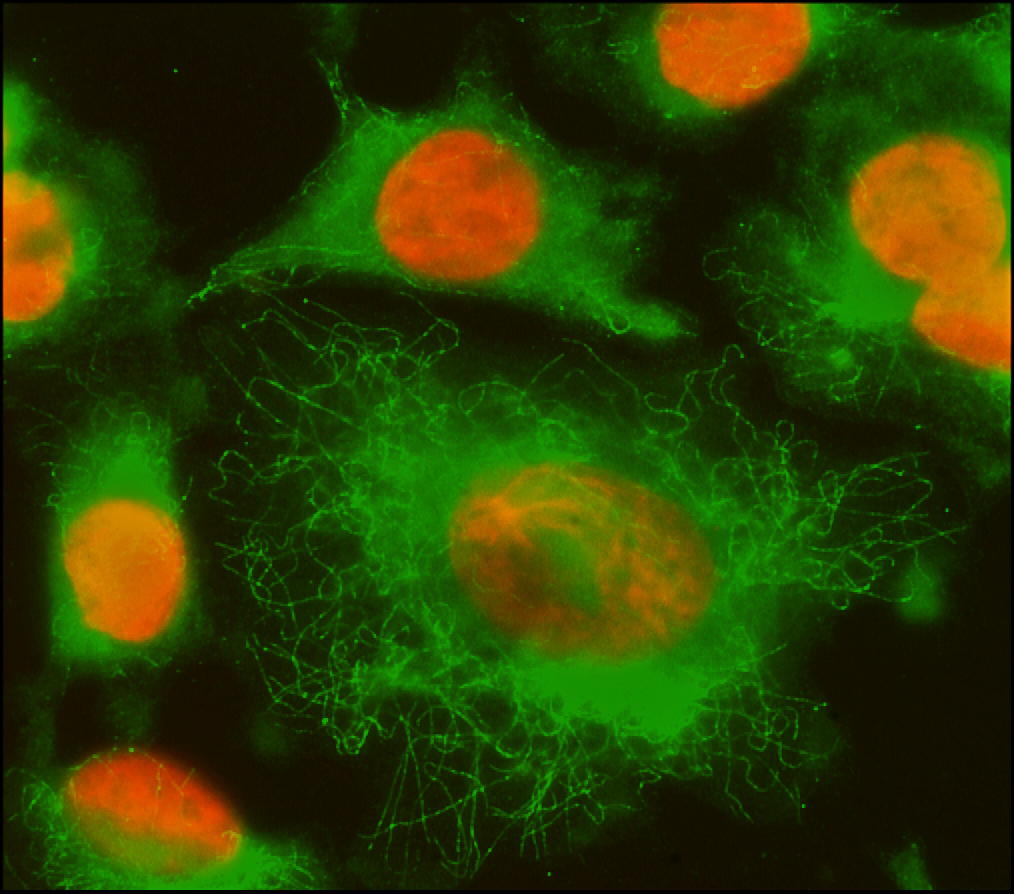

Supplement: S1 File — (ZIP) [file pone.0182022.s008.zip › 203_5IC50.jpg]

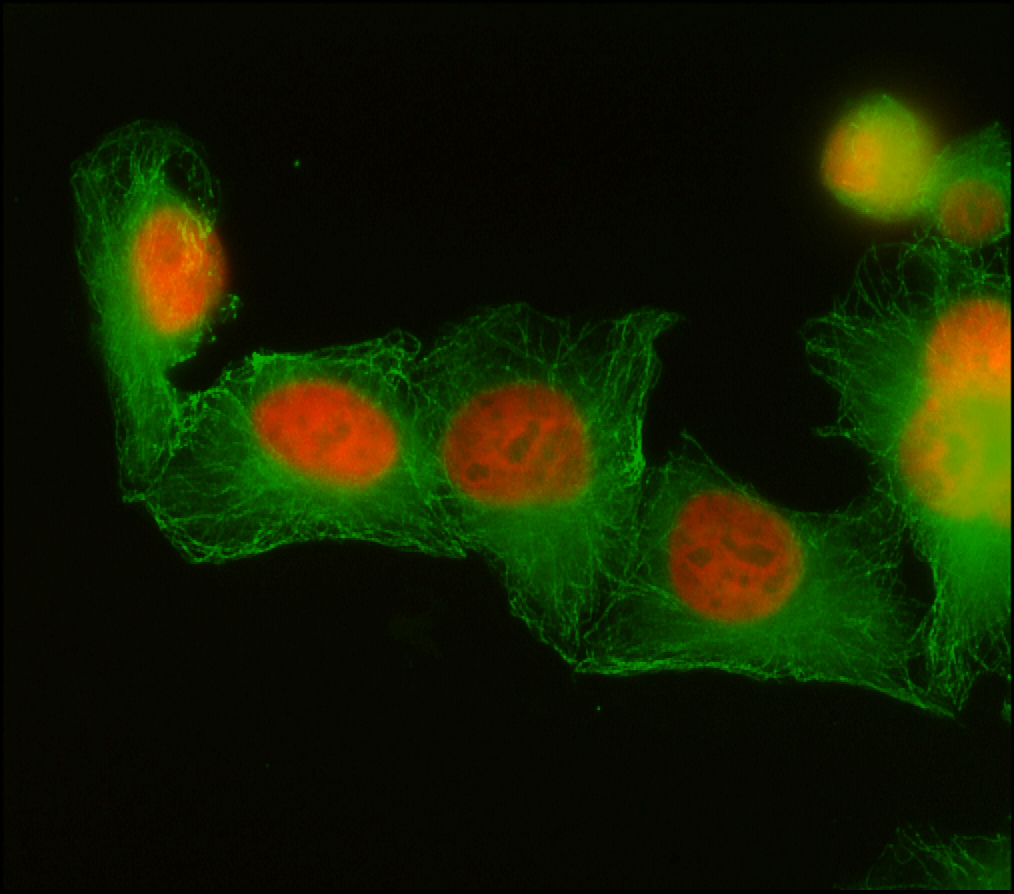

Supplement: S1 File — (ZIP) [file pone.0182022.s008.zip › 503_2IC50.jpg]

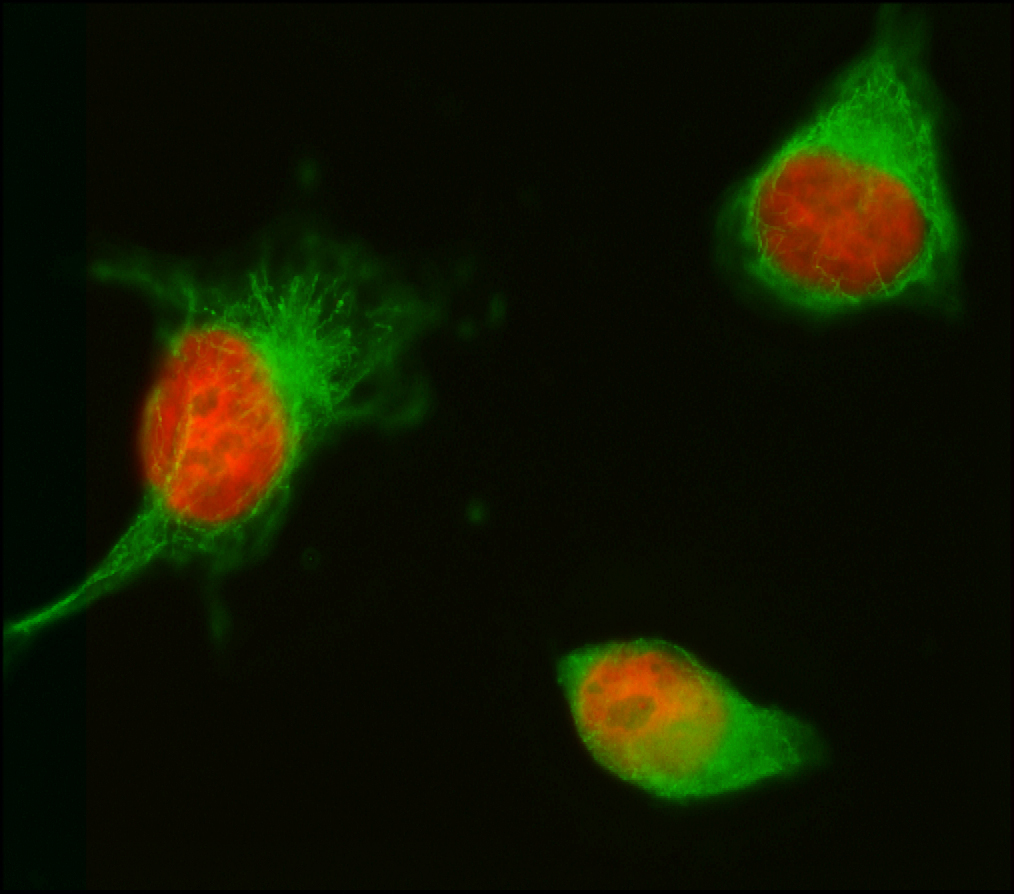

Supplement: S1 File — (ZIP) [file pone.0182022.s008.zip › 503_5IC50.jpg]

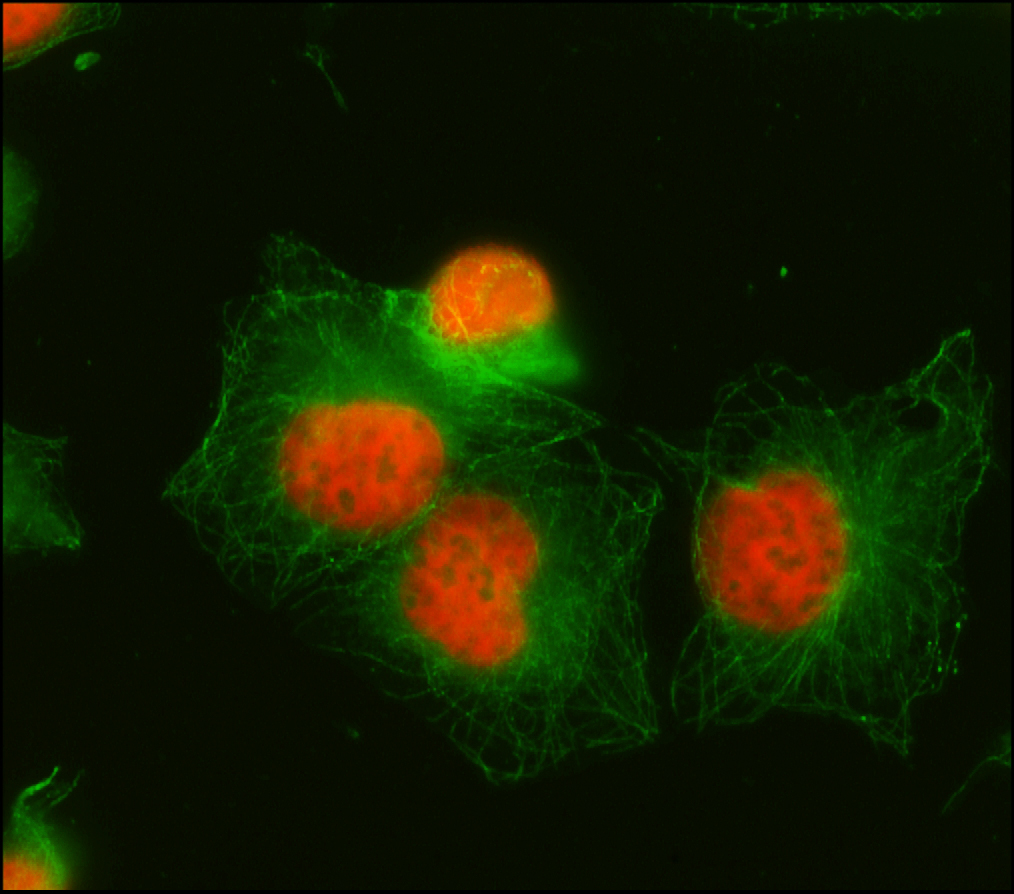

Supplement: S1 File — (ZIP) [file pone.0182022.s008.zip › Imiquimod_2IC50.jpg]

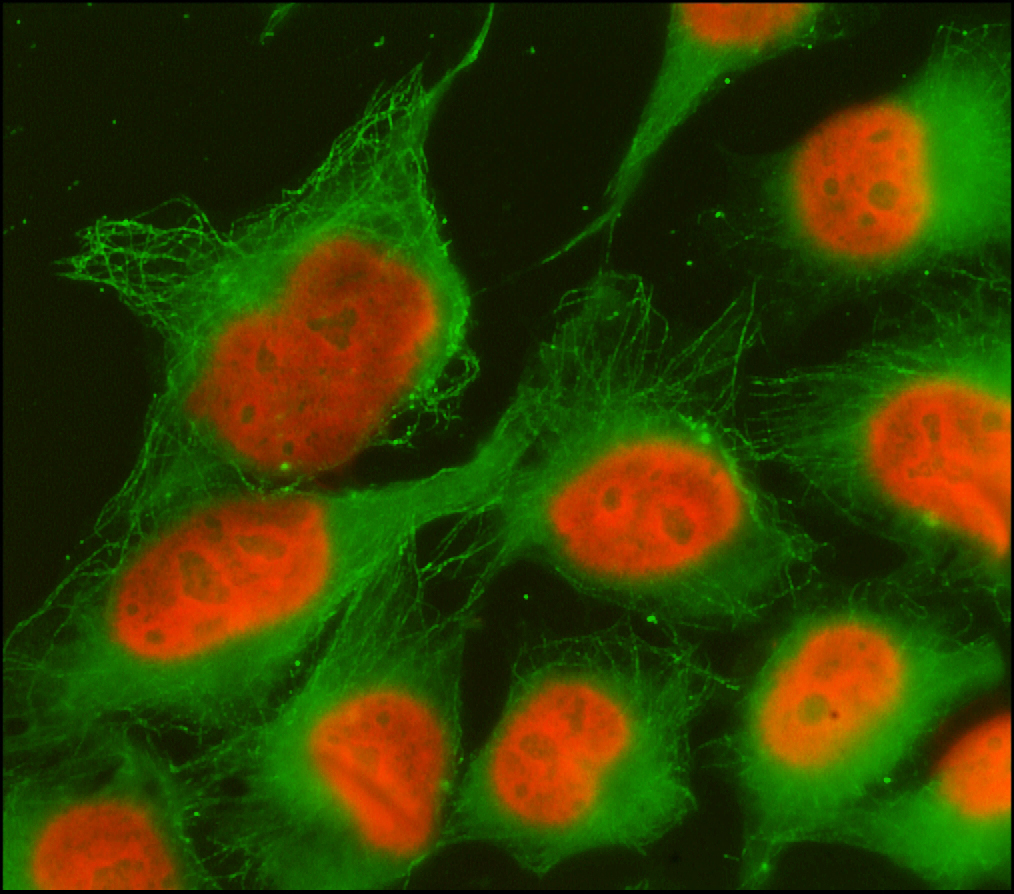

Supplement: S1 File — (ZIP) [file pone.0182022.s008.zip › Imiquimod_5IC50.jpg]

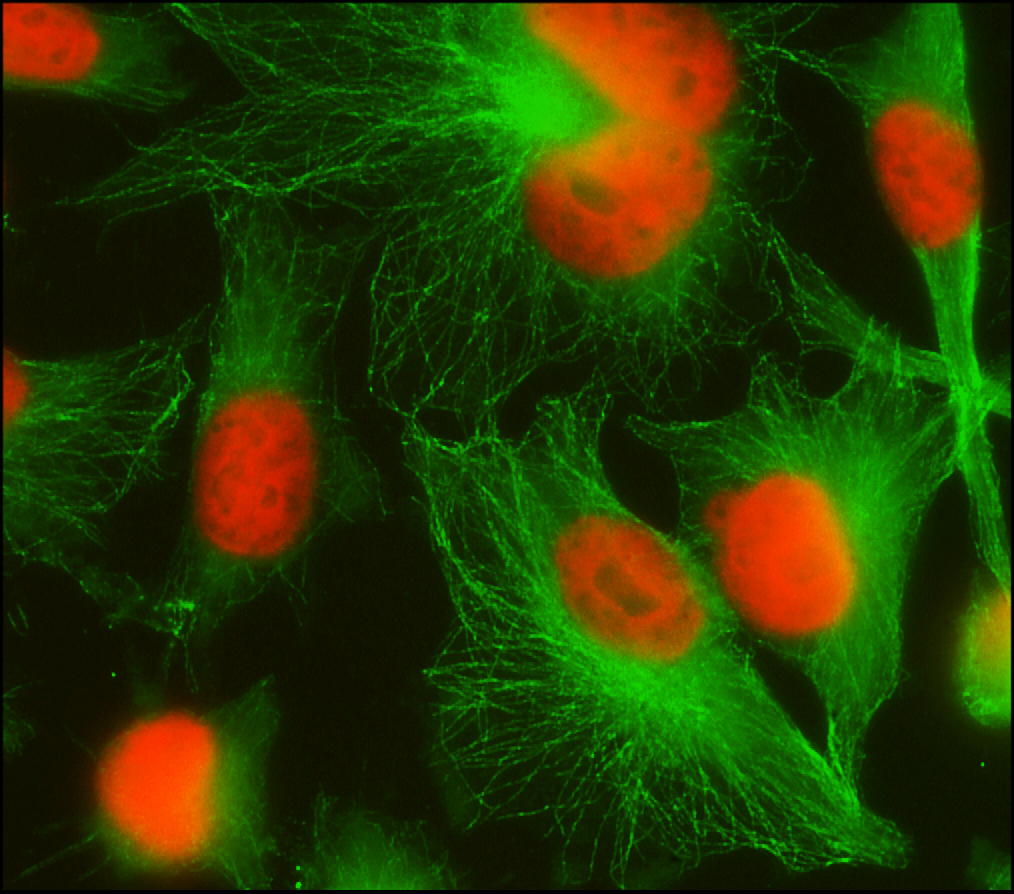

Supplement: S1 File — (ZIP) [file pone.0182022.s008.zip › NT_NT.jpg]
